# Quantitative phosphoproteomic analysis of acquired cancer drug resistance to pazopanib and dasatinib

**DOI:** 10.1016/j.jprot.2017.08.015

**Published:** 2018-01-06

**Authors:** Simon Vyse, Frank McCarthy, Malgorzata Broncel, Angela Paul, Jocelyn P. Wong, Amandeep Bhamra, Paul H. Huang

**Affiliations:** a Division of Cancer Biology, The Institute of Cancer Research, London SW3 6JB, UK; b Proteomics Core Facility, The Institute of Cancer Research, London SW3 6JB, UK

**Keywords:** Phosphoproteomics, Kinase inhibitors, Drug resistance, Pazopanib, Dasatinib, Cell signalling

## Abstract

Acquired drug resistance impacts the majority of patients being treated with tyrosine kinase inhibitors (TKIs) and remains a key challenge in modern anti-cancer therapy. The lack of clinically effective therapies to overcome resistance represents an unmet need. Understanding the signalling that drives drug resistance will facilitate the development of new salvage therapies to treat patients with secondary TKI resistance. In this study, we utilise mass spectrometry to characterise the global phosphoproteomic alterations that accompany the acquisition of resistance to two FDA-approved TKIs, pazopanib and dasatinib, in the A204 rhabdoid tumour cell line. Our analysis finds that only 6% and 9.7% of the quantified phosphoproteome is altered upon the acquisition of pazopanib and dasatinib resistance, respectively. Pazopanib resistant cells display elevated phosphorylation in cytoskeletal regulatory pathways while dasatinib resistant cells show an upregulation of the insulin receptor/IGF-1R signalling pathway. Drug response profiling rediscovers several previously reported vulnerabilities associated with pazopanib and dasatinib resistance and identifies a new dependency to the second generation HSP90 inhibitor NVP-AUY-922. This study provides a useful resource detailing the candidate signalling determinants of acquired TKI resistance; and reveals a therapeutic approach of inhibiting HSP90 function as a means of salvage therapy to overcome pazopanib and dasatinib resistance.

**Significance:**

Pazopanib and dasatinib are tyrosine kinase inhibitors (TKIs) approved for the treatment of multiple cancer types. Patients who are treated with these drugs are prone to the development of drug resistance and consequently tumour relapse. Here we use quantitative phosphoproteomics to characterise the signalling pathways which are enriched in cells that have acquired resistance to these two drugs. Furthermore, targeted drug screens were used to identify salvage therapies capable of overcoming pazopanib and dasatinib resistance. This data advances our understanding of the mechanisms of TKI resistance and highlights candidate targets for cancer therapy.

## Highlights

• Pazopanib resistant cells display elevated phosphorylation in cytoskeletal regulatory pathways.
• Phosphoproteins in the insulin and IGF-1R pathways are upregulated in dasatinib resistant cells.
• Less than 10% of the phosphoproteome is altered in acquired drug-resistant A204 cells.
• Both dasatinib and pazopanib resistant A204 cells are vulnerable to HSP90 inhibition.

## Graphical abstract

**Figure:**
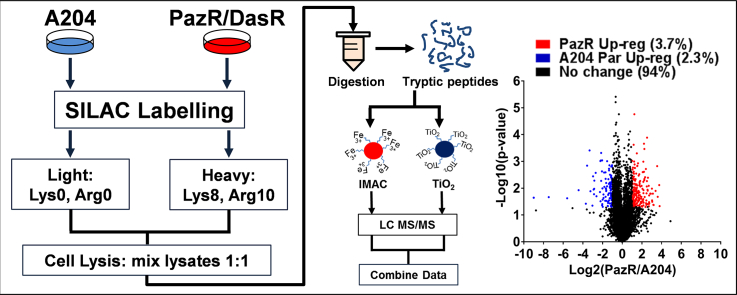

## 1 Introduction

Tyrosine kinase inhibitors (TKIs) have emerged as a major class of anti-cancer agents that display efficacy in a range of tumour types including lung cancer, chronic myeloid leukaemia (CML) and gastrointestinal stromal tumours (GIST) [1], [2]. However efficacy is often short-lived with the majority of patients going on to develop acquired resistance and tumour recurrence after prolonged drug treatment [3]. Studies in cell line models have revealed several major mechanisms of resistance that have been clinically observed, including the acquisition of drug resistant mutations in the target kinase, activation of bypass signalling pathways and phenotypic alterations such as epithelial-mesenchymal-transition (EMT) [3], [4], [5], [6]. These drug resistant cells arise either from selection of pre-existing clones within a heterogeneous tumour cell population or through the adaptation and subsequent evolution of drug-tolerant persister cells [7], [8]. Given that most patients who progress on TKI treatment have limited options for subsequent lines of therapy, there is an urgent need to develop effective salvage therapies to treat patients whose tumours relapse as a result of acquired drug resistance.

Pazopanib and dasatinib are multi-target TKIs that inhibit a distinct but overlapping spectrum of tyrosine kinases [9], [10], [11], [12]. Pazopanib is approved for advanced soft tissue sarcoma and renal-cell carcinoma [13], [14] while dasatinib is licensed for the treatment of CML and Philadelphia chromosome-positive acute lymphoblastic leukaemia (ALL) [15], [16]. Of note, the mechanisms of acquired resistance to pazopanib are poorly characterised in part because there are very few cell line models that harbour intrinsic sensitivity to this drug [17]. Despite the largely distinct target selectivity profiles of these two drugs, we have recently demonstrated that in the context of the SMARCB1-deficient rhabdoid tumour cell line A204, acquired resistance to these two compounds is associated with the downregulation of a common target PDGFRα [12]. This acquired resistance could be overcome by the inhibition of bypass signalling initiated by the FGFR1 kinase with inhibitors such as BGJ398, AZD4547 and ponatinib as salvage therapy [12].

Although our laboratory was able to identify common molecular alterations in PDGFRα and FGFR1 in the dasatinib- and pazopanib-resistant A204 cell lines, gene expression and copy number analyses of these cells have revealed clear differences between their molecular profiles [12]. For instance, the dasatinib-resistant cells harboured additional gains on chromosome 17 and losses in chromosome 13 which were not observed in the pazopanib-resistant line [12]. These differences suggest that there are likely to be additional dependencies associated with acquired resistance to dasatinib and pazopanib which can be exploited for cancer therapy. Furthermore, the phosphotyrosine (pTyr)-based proteomics employed in our previous study was only able to identify < 5 tyrosine phosphorylated proteins that were upregulated in the two TKI resistant cell lines [12], limiting our ability to determine the signalling pathways enriched as a result of acquired drug resistance. The lack of significantly upregulated pTyr-containing proteins raises the possibility that the major alterations associated with drug resistance in the A204 cells may instead be driven by phosphoserine (pSer) and phosphothreonine (pThr) signalling events.

In this study we employ a global phosphoproteomics analysis strategy to identify pSer/pThr signalling alterations enriched in the pazopanib- (PazR) and dasatinib-resistant (DasR) A204 cell lines. In addition, we perform a targeted drug profiling analysis to determine new vulnerabilities associated with pazopanib and dasatinib resistance in these cells; with the goal of identifying additional salvage therapy candidates to treat patients who have acquired resistance to these drugs. Phosphoproteomics has been extensively used to reveal signalling pathways driving resistance to multiple TKIs including the approved drugs erlotinib, lapatinib, imatinib and sorafenib among others [18], [19], [20], [21], [22], [23]. More recently, the value of utilising small panels of targeted drugs directed against key regulators of cancer cell survival to screen for combinations to overcome acquired drug resistance has been successfully demonstrated in lung cancer [24]. Here we utilise these two approaches to determine the signalling pathways which are enriched in pazopanib- and dasatinib-resistant cells and uncover a new vulnerability to the HSP90 inhibitor NVP-AUY-922 which has utility in overcoming acquired resistance to these TKIs.

## 2 Methods

### 2.1 Cell culture and derivation of acquired resistant sublines

Cells were cultured in DMEM media supplemented with 10% FBS, 2 mM glutamine, 100 units/ml penicillin and 100 mg/ml streptomycin in 95% air, 5% CO_2_ atmosphere at 37 °C. For SILAC experiments, A204 cells and resistant sublines were cultured in SILAC DMEM media (Thermo Fisher Scientific) supplemented with light lysine and arginine (R0K0) (Sigma) and heavy lysine and arginine (R10K8) (Goss Scientific), respectively. To generate resistant sublines, A204 cells were grown initially in DMEM media containing dasatinib and pazopanib (LC laboratories) at a concentration of 500 nM [12]. The drug was incremented when the cells had proliferated to near confluency alongside minimal visible cell death. Drug concentration was incremented from 2 μM to 3 μM and 5 μM in a stepwise manner over 6 weeks. A final drug concentration of 5 μM was maintained in resistant cells. Media and drug were replenished twice weekly.

### 2.2 Cell viability assays

Cells (2000/well) were seeded in a 96-well plate and treated with inhibitors at the indicated doses for 72 h prior to assessment of cell viability using Cell Titre Glo (Promega), following the manufacturer's recommendations. IC_50_ data were generated from dose-response curves fitted using a four-parameter regression fit in GraphPad Prism 6 software. Inhibitors used in this study include Gefitinib, Rociletinib, Lapatinib, Neratinib, Sorafenib, Ceritinib, Crizotinib, Pazopanib, Sunitinib, Dasatinib, Ponatinib, AZD4547, Bosutinib, BEZ235, Trametinib, NVP-AUY-922, Imatinib (LC laboratories) AZD9291, PF-562271, Palbociclib, BGJ398, MK2206, AZD5363 (Selleck Chemicals), BX-795, MRT67307 (Sigma-Aldrich), JQ1 (Cayman Chemical Company), DDR1-in-1 (Tocris), CCT244747 (ICR).

### 2.3 Colony formation assays

Cells were seeded at low density (10,000/well) in 6 well plates and after 24 h were treated with inhibitors at the indicated doses for a duration of 2 weeks. Media containing inhibitors was replenished every 72 h. Following this, cells were fixed using Carnoy's Fixative (3:1 methanol: acetic acid) and stained with 1% crystal violet solution (Sigma-Aldrich).

### 2.4 Phosphoproteomic enrichment and sample preparation

Phosphoproteomic analysis was performed as previously described [25] with the following modifications: SILAC labelled cells (biological triplicates) were lysed in 8 M urea and equal amounts of heavy (DasR or PazR cells) and light (parental cells) lysates were mixed prior to reduction, alkylation and trypsin digestion. Peptides were desalted on a C18 cartridge, eluted with 25% acetonitrile and lyophilised to dryness. The sample was reconstituted with 400 μl of IP buffer (100 mM Tris, 100 mM NaCl, 0.3% NP-40, pH 7.4) and the pH was adjusted to 7.4. After immuno-precipitation with pTyr100, pTyr1000 (Cell Signaling Technology) and 4G10 (Merck Millipore) for the phosphotyrosine-containing peptides, which were used in a prior study [12], the supernatant was subjected to phosphopeptide enrichment. 2 mg of cell lysate from the supernatant was enriched for phosphopeptides using sequential immobilised metal affinity chromatography (IMAC) on FeCl_3_ charged NTA beads as previously described [25].

A further 2 mg of cell lysate from the supernatant was separately enriched for phosphopeptides using TitanSphere Phos-TiO_2_ spin tips (GL Sciences). Spin tips were conditioned using 2 × 20 μl 80% acetonitrile/0.4% trifluoroacetic acid solution, followed by equilibration with 20 μl 60% acetonitrile/0.3% trifluoroacetic acid/25% lactic acid. Tips were spun at 3000 ×* g* for 2 min between each conditioning or equilibration step. The starting peptide sample was vacuum dried and reconstituted in 50 μl 0.1% trifluoroacetic acid solution. The reconstituted sample was mixed with 150 μl 60% acetonitrile/0.3% trifluoroacetic acid/25% lactic acid, added to an equilibrated spin tip and spun at 1000 x g for 10 mins. The flow through was collected and applied an additional two more times to the same spin tip to enhance adsorption of phosphopeptides. Following this, the flow through was then applied to a new spin tip and the same enrichment process was followed and analysed separately. After binding of phosphopeptides, spin tips were rinsed twice with 20 μl 60% acetonitrile/0.3% trifluoroacetic acid/lactic acid and five times with 20 μl of 80% acetonitrile/0.4% trifluoroacetic acid and spun at 3000 ×* g* for 2 min between each step. Phosphopeptides were eluted using 2 × 50 μl of 5% NH_4_OH solution and 1 × 50 μl pyrrolidine. Eluates were combined and vacuum dried before LC-MS/MS analysis.

### 2.5 Liquid Chromatography-Tandem Mass Spectrometry (LC-MS/MS)

For IMAC-enriched samples, reversed phase chromatography was performed on eluted peptides using a Dionex UltiMate 3000 RSLC nano system (Thermo Fisher Scientific). The phosphopeptide-enriched eluates were analysed as 6 μl injections, and loaded on to a Acclaim PepMap100 C18 trap cartridge trap cartridge at 8 μl/min 2% acetonitrile/0.1% trifluoroacetic acid (0.5 mm i.d. × 5 mm, 5 μm bead size, 100 Å pore size; loaded in a bi-directional manner). Peptides were then resolved on a 75 μm I.D. 15 cm C18 packed emitter column (3 μm particle size; NIKKYO TECHNOS CO.,LTD). Phosphopeptide-enriched samples were run over 125 min using a three-step gradient of 96:4 to 65:35 buffer A:B (*t* = 0 min 4% B, 5 min 4% B, 14 min 10% B, 118 min 35% B, 125 min 50% B) at 250 nl/min. Peptides were ionised by electrospray ionisation using 1.8 kV applied immediately pre-column via a microtee built into the nanospray source. Sample was infused into an LTQ Velos Orbitrap mass spectrometer (Thermo Fisher Scientific) directly from the end of the tapered tip silica column (6–8 μm exit bore). The ion transfer tube was heated to 275 °C and the S-lens set to 60%. MS/MS were acquired using data dependent acquisition based on a full 30,000 resolution FT-MS scan with preview mode disabled and no internal lock mass was used. The top 10 most intense ions were fragmented using enhanced ion trap scans. Precursor ions with unknown or single charge states were excluded from selection. Automatic gain control was set to 1,000,000 for FT-MS and 30,000 for IT-MS/MS, full FT-MS maximum inject time was 500 ms and normalised collision energy was set to 35% with an activation time of 10 ms. Total lysate peptides were subjected to wideband activation to co-fragment precursor ions undergoing neutral loss of up to − 20 *m*/*z* from the parent ion, including loss of water/ammonia. Multistage activation (MSA) was used to target phosphoserine/threonine peptides by fragmenting precursor ions undergoing neutral loss of 32.70, 49.00, 65.40 and 98.00 *m*/*z*, corresponding to neutral loss of phosphate, if observed in the top 3 most intense fragment ions. MS/MS was acquired for selected precursor ions with a single repeat count acquired after 8 s delay followed by dynamic exclusion with a 10 ppm mass window for 45 s based on a maximal exclusion list of 500 entries.

The equivalent of 2 μg of total lysate was also run according to the above conditions to measure the total proteome for subsequent normalisation of phosphoproteomic data. The total lysates were run over 245 min using a three-step gradient of 96:4 to 65:35 buffer A:B (*t* = 0 min 4% B, 5 min 4% B, 45.0 min 10% B, 230.0 min 35% B, 245.0 min 50% B) and the top 20 most intense ions were fragmented by collision-induced dissociation and analysed using normal ion trap scans as described above.

For TiO_2_-enriched samples, peptides were resolved on a 75 μm I.D. 50 cm C18 Easy-Spray packed emitter column (2 μm particle size; PepMap RSLC, Thermo Fisher Scientific) over 240 min using a multi-step gradient of buffers A:B (*t* = 0 min 5% B, *t* = 5.5 min 4% B, *t* = 45 min 10% B, *t* = 175 min 25% B, *t* = 245 min 50% B, *t* = 250 min, 95% B, *t* = 255 min, 95% B, *t* = 260 min 4% B, *t* = 280 4% B) (buffer A: 2% acetonitrile/0.1% formic acid; buffer B: 80% acetonitrile/0.1% formic acid) at 250 nl/min. Peptides were ionised by electrospray ionisation using 2.3 kV applied using the Easy-Spray ion Source. Sample was infused into a Q-Exactive HF mass spectrometer (Thermo Fisher Scientific) directly from the packed emitter (5 μm exit bore). The ion transfer tube was heated to 275 °C and the S-lens set to 50%. MS/MS were acquired using data dependent acquisition based on a full FT-MS scan from 350 to 1850 *m*/*z* at 120,000 resolution, with a target Automatic Gain Control (AGC) value of 3,000,000 and a maximum injection time of 50 ms. No internal lock mass calibrant was used. The top 15 most intense ions were fragmented by higher energy collision-induced dissociation (HCD) and dynamically excluded for 30 s. The normalised collision energy was set to 32 with an activation time of 10 ms. Precursor ions with unknown or single charge states were excluded from selection. Fragmented ions were scanned in the FT-Orbitrap at 60,000 resolution (selected first mass at 100 *m*/*z*) with a target AGC value of 50,000 and a maximum injection time of 100 ms.

### 2.6 Data analysis

The data were processed with MaxQuant [26] (version 1.5.5.1) and the peptides were identified (maximal mass error = 6 ppm and 20 ppm for precursor and product ions, respectively) from the MS/MS spectra searched against human UniProt database using Andromeda [27] search engine. The following peptide bond cleavages: arginine or lysine followed by any amino acid (a general setting referred to as Trypsin/P) and up to two missed cleavages were allowed. SILAC based experiments in MaxQuant were performed using the built-in quantification algorithm [26] with minimal ratio count = 2 and enabled ‘Requantify’ feature. For each of the three biological replicate experiments, two technical replicates of the IMAC-phosphopeptide enrichment; two technical replicates of the TiO_2_-phosphopeptide enriched samples; and three technical replicates of the total proteome were analysed. Cysteine carbamidomethylation was selected as a fixed modification whereas methionine oxidation; deamidation of asparagine and glutamine; glutamine to pyro-glutamic acid; acetylation of protein N-terminus; with phospho (STY) as variable modifications for phosphoproteome searches. The false discovery rate was set to 0.01 for peptides, proteins and sites. Other parameters were used as default in the software. “Unique and razor peptides” mode was selected to allow identification and quantification of proteins in groups. Data were further analysed using Microsoft Office Excel 2010 and Perseus [28] (version 1.5.5.3). Both phosphoproteomic and proteomic data were filtered to remove potential contaminants and IDs originating from reverse decoy sequences. Proteomic data was also filtered to exclude proteins only identified by site. To account for deviations from a 1:1 mix of heavy:light starting material, the median H/L ratio across the entire proteome dataset was used to normalize the phosphoproteomic dataset. The log_2_ values of the H/L ratios were then determined. Phosphorylation sites (STY) were filtered to include only high confidence phosphosite IDs (localization probability ≥ 75%). The dataset was then filtered for only valid quantifiable IDs in at least two out of three biological replicates. The mass spectrometry proteomics data have been deposited to the ProteomeXchange Consortium via the PRIDE [29] partner repository with the dataset identifier PXD005536.

### 2.7 Bioinformatic analysis

Biological replicate overlap and phosphorylated amino acid distribution were analysed within Perseus (1.5.5.3) [28]. The phosphoproteome dataset was then annotated with the PhosphositePlus known sites database [30]. The online tool Venny 2.1 (http://bioinfogp.cnb.csic.es/tools/venny/) was used to generate Venn diagrams and GraphPad Prism 7.02 was used to generate the pie charts.

One sample *t*-tests were performed on SILAC log_2_ ratios to determine significantly different regulated phosphosites; where the null hypothesis was that the phosphopeptide abundances were unchanged and the log_2_ SILAC ratio was equal to 0. Those phosphosites that were either two-times up-regulated in the A204 parental (*t*-test difference < − 1) or up-regulated in the PazR or DasR (*t*-test difference > 1) cells and significantly different (*p* < 0.05) were analysed for enrichment. These data are presented as volcano plots generated in GraphPad Prism 7.02 where the statistical significance (*p* < 0.05) was –log_10_ transformed (y-axis) and plotted against the *t*-test difference (x-axis).

Enrichment analysis was performed using DAVID Bioinformatics Resources 6.8 [31] with human genome as a background dataset. KEGG (Kyoto encyclopedia of genes and genomes) [32], Uniprot keyword and sequence feature categories [33], Interpro protein function analysis [34], SMART (Simple Modular Architecture Research Tool) protein domain [35] and COG (Clusters of Orthologous Groups) Analysis Ontology [36] annotation databases were used for analysis. Protein annotation enrichment analysis of the phosphoproteome dataset was performed using the DAVID functional annotation tool and a modified Fisher Exact Test called EASE (Expression Analysis Systematic Explorer) score, comparing up-regulated phosphorylated proteins of PazR and DasR with their corresponding up-regulated phosphorylated proteins in the A204 parental cell line. A statistical cut off of 0.005 was applied. Multiple hypothesis testing was controlled using a Benjamini-Hochberg FDR threshold of 0.1. An intersection size of 3 or more was considered to be enriched. A bar chart of the data was then generated within GraphPad Prism 7.02. Additionally, the DAVID enrichment analysis was subjected to network mapping for visualisation using the application EnrichmentMap 2.2.1 within the Cytoscape 3.4.0 software [37]. Lists of phosphoproteins from enrichment clusters were generated and further investigated using the online application STRING 10.5 [38] to construct protein networks and analyse their associations. If necessary, 5 additional STRING interactors were imputed to the networks to propose possible intact, but not measured, systems. The network images were generated from the STRING output of proteins and their interaction score using Cytoscape 3.4.0.

For drug screen analysis, clustering was performed and heat maps generated within Perseus as described above across each dose of drug (100 or 500 nM) and cell line (A204 parental, DasR and PazR) using cell viability values normalised to DMSO control (*n* = 2 or 3).

## 3 Results

### 3.1 Characterisation of the phosphoproteome in parental and acquired resistant A204 cells

**Fig. 1:**
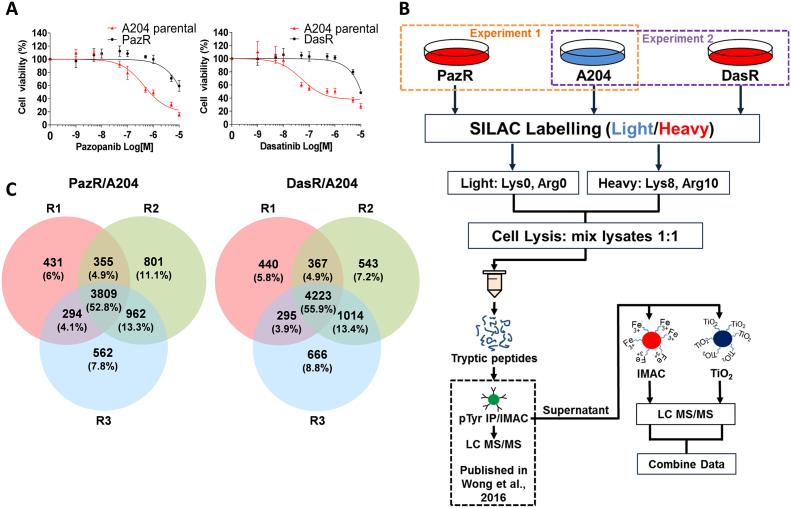
Experimental outline and phosphoprotome dataset overview. (A) Dose response curve of A204 parental and PazR cells to pazopanib and A204 parental and DasR cells to dasatinib respectively. (B) Schematic of sample preparation workflow. Pazopanib and dasatinib resistant A204 cell lines (PazR & DasR respectively) were generated and heavy SILAC labelled as previously described [12]. A204 parental cells were light SILAC labelled. After cell lysis, either heavy PazR or DasR were mixed 1:1 with light A204 parental lysate then reduced, alkylated and trypsin digested. The resulting peptides underwent phospho-tyrosine (pTyr) peptide immunoprecipitation, data previously published [12]. The supernatant from the immunoprecipitation was further enriched with immobilised metal affinity chromatography (IMAC) or titanium dioxide (TiO_2_) prior to liquid chromatography tandem mass spectrometry analysis (LC MS/MS). (C) Venn diagrams show distribution of phosphorylation sites across three biological replicates (R1, R2 and R3) in PazR/A204 and DasR/A204 experiments.

Pazopanib resistant (PazR) and dasatinib resistant cells (DasR) were previously derived from the A204 parental cell line by long-term escalating dose treatment with drug [12] (Fig. 1A). Briefly, A204 cells were initially grown in media containing 500 nM of pazopanib or dasatinib and the drug dose increased when the cells proliferated to near confluency alongside minimal visible cell death. Drug concentration was then increased from 2 μM to 3 μM and then 5 μM in a stepwise manner over 6 weeks. A final drug concentration of 5 μM was maintained in resistant cells. We subjected the cell lines to stable isotope labelling with amino acids in cell culture (SILAC) with the PazR and DasR cells being ‘heavy labelled’ and the parental A204 cell line being ‘light labelled’ (Fig. 1B). Cells were lysed, combined in a 1:1 ratio and lysates digested with trypsin. We have performed an analysis of the pTyr phosphoproteome of these cells using phosphopeptide immunoprecipitation of the SILAC labelled cell lysates in a previously reported study [12]. In this current study, the supernatant from this pTyr immunoprecipitation was subjected to either immobilised metal affinity chromatography (IMAC) or titanium dioxide (TiO_2_) phosphopeptide enrichment prior to single-shot liquid chromatography tandem mass spectrometry (LC-MS/MS) in biological triplicates (Fig. 1B). The mass spectrometry data from both phosphopeptide enrichment strategies were combined and analysed together using the MaxQuant algorithm [26].

Collectively, we identified 7214 unique phosphorylation sites on 2372 proteins in the PazR/A204 comparison and 7548 unique phosphosites on 2494 proteins in the DasR/A204 comparison across all three biological replicates (Fig. 1C and Table S1, Table S2). In both sets of experiments, analysis of the distribution of phosphorylated residues shows the expected classical distribution of pSer:pThr:pTyr ratios (~ 90:10:1) as previously reported (Fig. S1A) [39]. We observed pTyr sites (~ 1% of all phosphosites) in the analysis despite prior pTyr phosphopeptide enrichment (Fig. S1A), indicating that immunoprecipitation did not deplete all the pTyr-containing peptides in the lysate. This may be the result of previously reported restricted pTyr motifs recognised by anti-phosphotyrosine antibodies used in the immunoprecipitation [40]. Consistent with this idea, a comparative analysis of the identified pTyr sites from the previous immunoprecipitation and the current IMAC/TiO2 enrichment shows the overlap of only 1 phosphorylation site between the two datasets (Fig. S2). Comparing our phosphoproteomic datasets with the PhosphoSitePlus database showed that 389 and 394 novel phosphosites were identified in the PazR/A204 and DasR/A204 experiments, respectively (Fig. S1B, Table S1, Table S2) [30]. The total number of phosphosites identified in our dataset is comparable with previous phosphoproteomic studies (ranging from 2000 to 5000 phosphosites) where single-shot sample injection into the mass spectrometer was carried out with no additional fractionation [41], [42], [43], [44], [45].

### 3.2 Quantitative phosphoproteomic analysis of pazopanib resistance

**Fig. 2:**
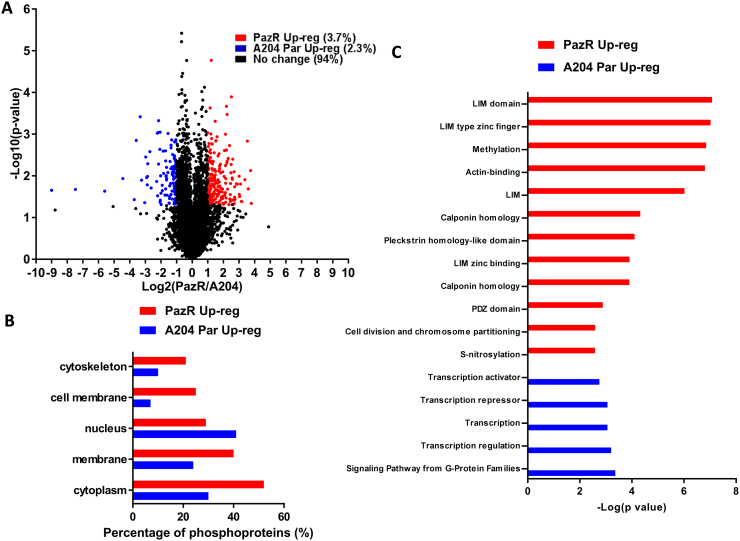
Phosphoproteomic profile of PazR versus A204 parental cells. (A) Volcano plot depicting the phosphoproteome of PazR versus A204 parental cells. All ratios were median-normalised and log_2_ transformed. A one sample *t*-test was performed where the null hypothesis was equal to 0. The statistical significance was –log_10_ transformed (y-axis) and plotted against the *t*-test difference (x-axis). Phosphosites that display at least 2-times increase in PazR (red) or increase in A204 parental (blue) with *p* < 0.05 are indicated. Legend shows percentage of phosphosites that were up-regulated in PazR cells or A204 parental cells as well as phosphosites that displayed no change between the two cell lines. (B) Uniprot keyword protein localisation annotation terms linked to either statistically significant PazR or A204 parental up-regulated phosphoproteins generated using the DAVID functional annotation tool [31]. (C) Annotation enrichment analysis of phosphoproteins up-regulated in either the PazR or A204 parental cells compared against the human genome using DAVID. The resultant *p* values of each term were –log_10_ transformed. Multiple hypothesis testing was controlled using a Benjamini-Hochberg FDR threshold of 0.1.

**Fig. 3:**
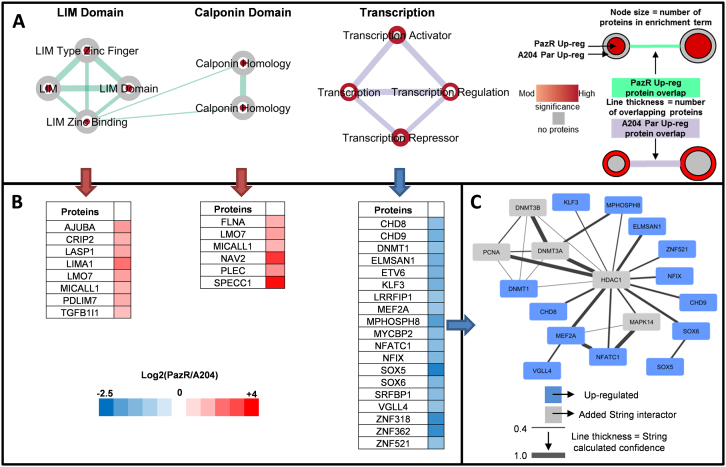
Biological function analysis of PazR versus A204 parental cells. (A) Annotation enrichment analysis of PazR and A204 parental up-regulated phosphoproteins using the DAVID functional annotation tool. Network maps represent clusters of annotation terms from different databases with associated function. Nodes represent each term and the connecting line their association; line thickness is number of overlapping proteins. The inner and outer nodes are PazR and A204 parental datasets, respectively. Node size represents the number of proteins annotated with that term. The colour intensity of the node represents the significance of enrichment (ranging from moderate to high) and grey depicts no proteins. (B) Heat map of proteins in network cluster based on the log_2_(PazR/A204) SILAC ratio. (C) An association network of proteins from the ‘transcription’ cluster analysed through the STRING application. Blue coloured proteins are from the cluster list and grey are added STRING interactors. Line thickness portrays the STRING calculated association confidence.

5420 phosphosites on 1950 proteins were quantified in two or more replicates in the PazR/A204 experiments (Fig. 2A). To determine the cellular localisation of phosphorylated proteins which are significantly upregulated in PazR or parental A204 cells, we interrogated our dataset using the Uniprot Keyword database and found that with the exception of the nucleus, phosphorylated proteins across multiple subcellular compartments were increased in PazR cells versus the parental A204 line (Fig. 2B). 198 phosphorylation sites on 112 proteins (3.7% of the phosphoproteomic dataset) were significantly upregulated > 2-times (> log_2_ +1) in PazR cells compared to parental A204 cells (Fig. 2A). These phosphoproteins that were upregulated in PazR cells were subjected to ontology enrichment analysis which revealed the enrichment of a number of ontology terms associated with cytoskeletal organisation (Fig. 2C). These included “actin-binding”, “LIM domain containing”, and “Calponin homology (CH) domain containing” proteins (Fig. 3A) [46], [47], [48], [49]. LIM domain-containing proteins comprise AJUBA, CRIP2, LASP1, LMP7, MICALL1, PDLIM7 and TGFB1l1 whilst CH-domain proteins include FLNA, LMO7, MICALL1, NAV2, PLEC and SPECC1 (Fig. 3B). This gene ontology enrichment analysis suggests that PazR cells upregulate multiple actin cytoskeletal-regulatory pathways which may play a role in maintaining its drug resistant state.

122 phosphosites on 71 proteins (2.3% of the dataset) were found to be significantly upregulated (< log_2_ –1) in the parental A204 cells compared to the PazR cells (Fig. 2A) with up to 40% being nuclear proteins (Fig. 2B). Ontology analysis of these phosphorylated proteins identified an enrichment of proteins involved in transcription regulation including the ontology terms “transcription regulation”, “transcription”, “transcription activator” and “transcription repressor” (Figs. 2C and 3A). These include the transcription factors ETV6, SOX5, SOX6, KLF3, NFIX and DNA binding proteins DNMT1, CDH8, CDH9 and VGLL4 (Fig. 3B). Upon interrogation with the STRING database [38], a subset of these proteins showed a well annotated protein-protein interaction network centred around the HDAC1 protein (Fig. 3C). The discovery that the phosphorylation of multiple transcription factors is upregulated in SMARCB1-deficient parental A204 rhabdoid tumour cells is consistent with the role of SMARCB1 in organising nucleosome structures surrounding transcriptional start sites in a genome-wide manner [50].

### 3.3 Quantitative phosphoproteomic analysis of dasatinib resistance

**Fig. 4:**
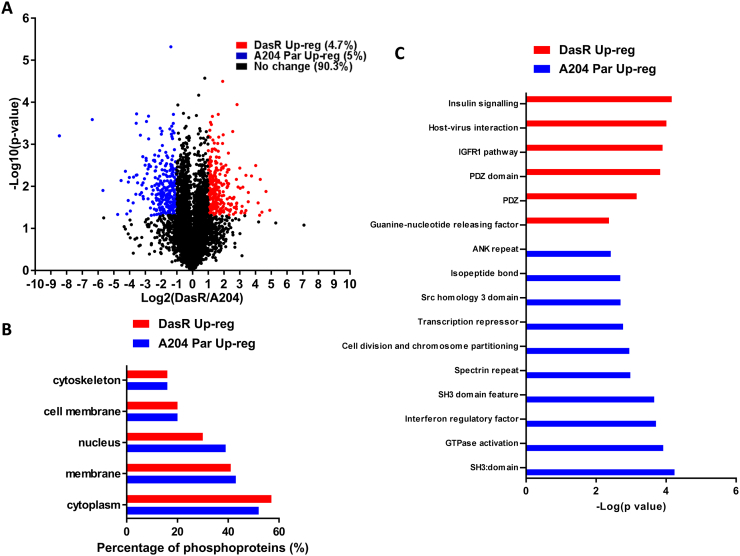
Phosphoproteomic profile of DasR versus A204 parental cells. (A) Volcano plot depicting the phosphoproteome of DasR versus A204 parental cells. All ratios were median-normalised and log_2_ transformed. A one sample *t*-test was performed where the null hypothesis was equal to 0. The statistical significance was –log_10_ transformed (y-axis) and plotted against the *t*-test difference (x-axis). Phosphosites that display at least 2-times increase in DasR (red) or increase in A204 parental (blue) with *p* < 0.05 are indicated. Legend shows percentage of phosphosites that were up-regulated in DasR cells or A204 parental cells as well as phosphosites that displayed no change between the two cell lines. (B) Uniprot keyword protein localisation annotation terms linked to either statistically significant DasR or A204 parental up-regulated phosphoproteins generated using the DAVID functional annotation tool [31]. (C) Annotation enrichment analysis of phosphoproteins up-regulated in either the DasR or A204 parental cells compared against the human genome using the DAVID application. The resultant *p* values of each term were –log_10_ transformed. Multiple hypothesis testing was controlled using a Benjamini-Hochberg FDR threshold of 0.1.

**Fig. 5:**
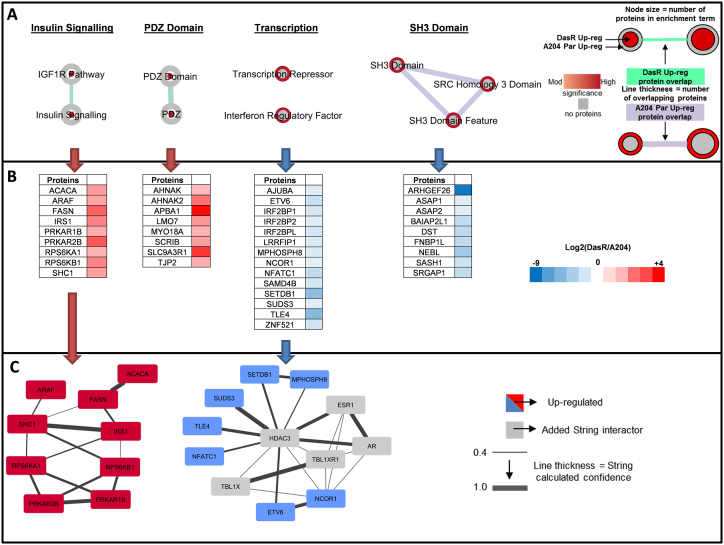
Biological function analysis of DasR versus A204 parental cells. (A) Annotation enrichment analysis of DasR and A204 parental up-regulated phosphoproteins using the DAVID functional annotation tool. Network maps represent clusters of annotation terms from different databases with associated function. Nodes represent each term and the connecting line their association; line thickness is number of overlapping proteins. The inner and outer nodes are DasR and A204 parental datasets, respectively. Node size represents the number of proteins annotated with that term. The colour intensity of the node represents the significance of enrichment (ranging from moderate to high) and grey depicts no proteins. (B) Heat map of proteins in network cluster based on the log_2_(DasR/A204) SILAC ratio. (C) An association network of proteins from the ‘insulin signalling’ and ‘transcription’ clusters were analysed through the STRING application. Red or blue coloured proteins are from the cluster lists and grey are added STRING interactors. Line thickness portrays the STRING calculated association confidence.

5899 phosphosites on 2086 proteins were quantified in two or more biological replicates in the DazR/A204 experiments (Fig. 4A). In contrast to the PazR/A204 dataset, both the DasR and parental A204 cell lines show comparable distribution of upregulated phosphorylated proteins across multiple cellular compartments (Fig. 4B). The exception is the nuclear compartment where the parental A204 cells have a slight increase in enrichment over the DasR cells. 279 phosphorylation sites on 157 proteins (4.7% of the dataset) were significantly upregulated > 2-times in DazR cells compared to parental A204 cells (Fig. 4A). Subjecting these upregulated phosphosites to gene ontology enrichment analysis (Fig. 4C) finds that the DasR cells shows a distinct spectrum of ontology terms compared to the PazR cells with the enrichment of insulin - and IGF-1R signalling pathway components and PDZ domain containing proteins. The insulin signalling pathway cluster includes the proteins ACACA, ARAF, FASN, IRS1, PRKAR1B, PRKAR2B, RPS6KA1, RPS6KB1 and SHC1 which together form a functional protein-protein interaction network (Fig. 5). PDZ domain containing proteins that are upregulated in DasR cells include proteins with a range of cellular functions such as cell migration regulation (AHNAK, AHNAK2, SCRIB), cytoskeletal and tight junction proteins (MYO18A and TJP2), and the sodium/hydrogen exchange cofactor SLC9A3R1 (Fig. 5A and B).

294 phosphorylation sites on 157 proteins (5% of the dataset) were found to be upregulated in the parental A204 versus the DasR cells (Fig. 4A). Enriched ontology terms include SH3 domain containing proteins (Fig. 4C) which play a role in small GTPase regulation and comprise key signalling proteins ARHGEF26, ASAP1, ASAP2, FNBP1L and SRGAP1 (Fig. 5A and B). Similar to the PazR/A204 dataset, there was an enrichment of transcriptional regulatory terms which include “transcription repressor” and “interferon regulatory factor” (Fig. 4C). These include the transcription factors ETV6, NFATC1, ZNF521 and transcriptional repressors NCOR1, TLE4 and SUDS3 (Fig. 5). A subset of these proteins feature as part of a protein-protein interaction network centred around the HDAC3 protein (Fig. 5C). The observation that protein-protein interaction networks involving the histone deacetylases (HDACs) are enriched in A204 parental cells in both the PazR/A204 and DasR/A204 experiments (Figs. 3C and 5C) is consistent with recent preclinical reports that HDAC inhibitors have therapeutic utility in reducing the proliferation of rhabdoid tumour cells including the A204 line [51], [52], [53].

### 3.4 Comparison of PazR and DasR phosphoproteomic datasets

**Fig. 6:**
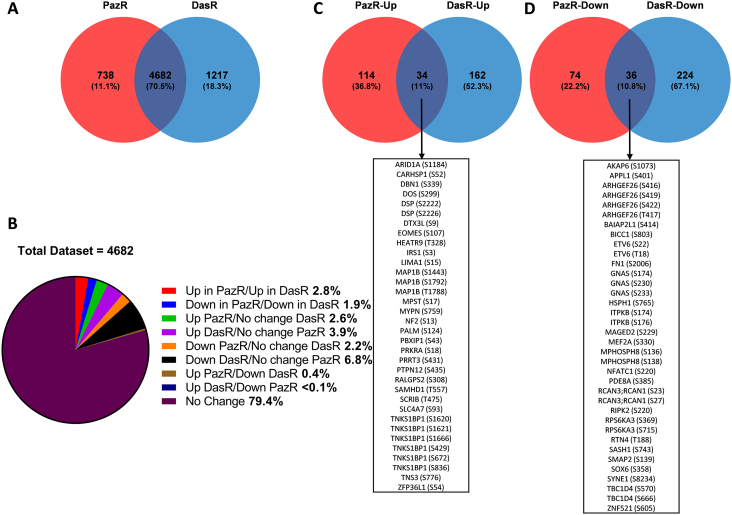
Comparative assessment of PazR and DasR cells. (A) Venn diagram to show overlap of phosphosites between the PazR and DasR datasets in at least 2 out of 3 biological replicates. (B) A pie chart distribution using only overlapping phosphosites of both PazR and DasR. Categories include: ‘Up’ (at least 2-times up-regulated versus parental), ‘down’ (at least 2-times down-regulated versus parental) and ‘no change’ (< 2-times up-regulated and < 2-times down-regulated). A statistical significance cut-off (*p*-value < 0.05) was then applied and the overlap between (C) up- or (D) down-regulated (at least 2-times) in PazR and DasR lines compared to A204 parental cells phosphorylation sites are shown.

A comparison between the two phosphoproteomic datasets revealed a 70.5% overlap with 4683 phosphorylation sites quantified across both resistant cell lines (Fig. 6A). Taken together, we find that 21.6% of the phosphoproteome is significantly altered upon the acquisition of secondary resistance in PazR and/or DasR sublines versus the parental A204 cells (Fig. 6B). Supporting our hypothesis that pazopanib and dasatinib induce different cellular reprogramming effects in the A204 cells, only 2.8% and 1.9% of observed phosphosites are similarly up- and down-regulated, respectively, in both datasets (Fig. 6B). The 34 upregulated and 36 downregulated phosphosites are detailed in Fig. 6C–D.

### 3.5 Drug response profiling identifies new vulnerabilities in drug resistant cells

Inspired by a recent targeted screen to identify drugs capable of overcoming bypass signalling pathways associated with acquired TKI resistance in lung cancer [24], we subjected both resistant lines and the parental A204 cells to short term treatment with a focused panel of 28 small molecule inhibitors at two different doses and measured cell viability. This panel comprised of kinase inhibitors targeting the major cellular signalling pathways important for cancer cell survival as well as inhibitors that target the BET bromodomain proteins (JQ1) and the HSP90 protein (NVP-AUY-922) which are currently in advanced clinical trials (Table S3).

**Fig. 7:**
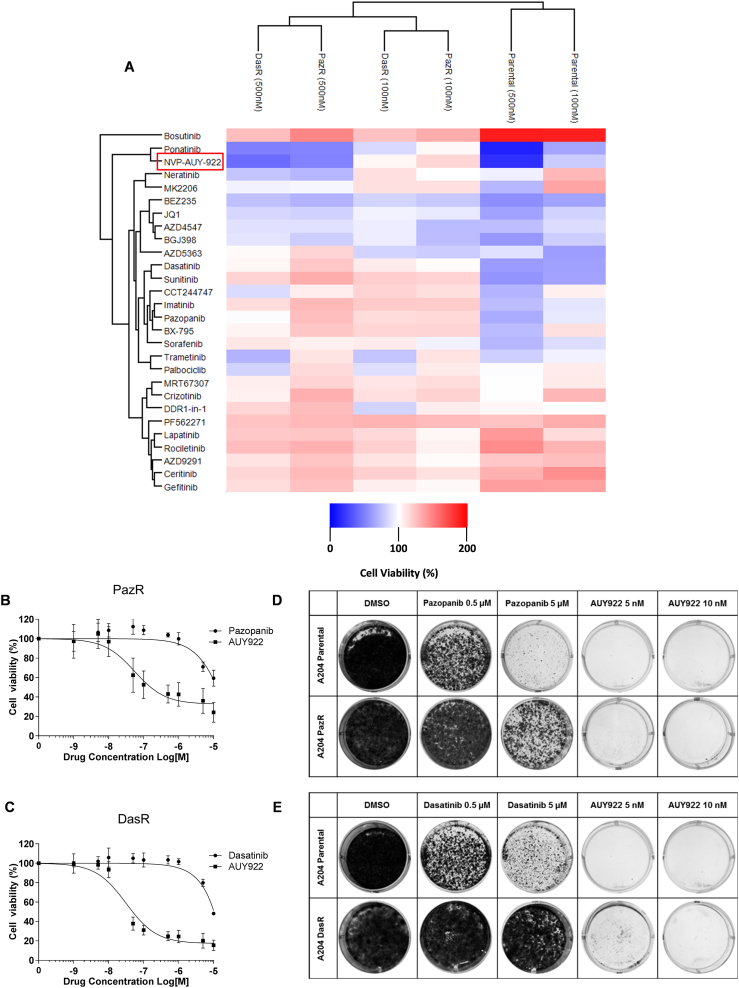
Drug profiling analysis of in A204 parental and resistant cell lines. (A) Heatmap depicting two-way hierarchical clustering of cell viability data in drug screen. A204 parental, pazopanib resistant and dasatinib cell lines were seeded in 96 well plates and viability was measured using Cell Titer Glo following 72 h of treatment with 28 small molecule inhibitors at 100 nM and 500 nM (or 10 nM and 50 nM for NVP-AUY-922). Two-way hierarchical clustering using Euclidean distance was performed. (B) Dose response curve of PazR cells to pazopanib or NVP-AUY-922 treatment. (C) Dose response curve of DasR cells to dasatinib or NVP-AUY-922 treatment. For (A), (B) and (C) cell viability is normalised to DMSO control and values represent mean ± SD (*n* = 2 or 3). Colony formation assays comparing (D) A204 parental and pazopanib resistant and (E) A204 parental and dasatinib resistant cell lines in the presence of drug. Cell lines were seeded at low density (10,000 cells/well) in a 6 well plate. After 2 weeks of treatment with inhibitors at the indicated doses, cells were fixed and colonies were stained using crystal violet for visualisation.

Two-way hierarchical clustering of the cell viability data demonstrates that the PazR and DasR cells share a more similar drug response profile compared to parental A204 cells (Fig. 7A). As shown in our previous study, the two resistant cell lines are highly sensitive to ponatinib treatment [12]. The screen also showed that the dual mTOR/PI3K inhibitor BEZ-235 sensitized both DasR and PazR which recapitulates the findings of a recent report on the use of this drug to overcome pazopanib resistance in patient-derived soft tissue sarcoma cells [54]. We also identify several inhibitors that are only effective in the DasR cells including the MEK inhibitor trametinib and to a lesser extent the CDK4/6 inhibitor palbociclib. MEK inhibitors have been shown to overcome drug resistance induced by the paradoxical activation of the MEK/ERK pathway through the weak binding of dasatinib to BRAF and CRAF [55]. The ability of our targeted screen to rediscover several previously identified vulnerabilities associated with pazopanib and dasatinib resistance provides confidence of the broad applicability of this strategy to identify salvage therapies to sensitize TKI-resistant cells.

This screen also uncovered a previously undescribed vulnerability of both PazR and DasR cells to the second generation HSP90 inhibitor NVP-AUY-922 which clustered together with ponatinib [56]. Dose response analysis confirms that PazR and DasR cells are sensitive to treatment with NVP-AUY-922 with IC_50_ values of 45.3 ± 14.3 nM and 28.4 ± 5.9 nM, respectively (Fig. 7B and C). Long-term colony formation assays show that low dose NVP-AUY-922 (5 nM) is capable of not only sensitizing both PazR and DasR cells but also killing parental A204 cells (Fig. 7D and E), suggesting that HSP90 inhibitors may be an effective option both as first-line and salvage therapy in rhabdoid tumours.

## 4 Discussion

This study is, to our knowledge, the first phosphoproteomic analysis of acquired resistance to pazopanib and dasatinib. We show that A204 cells that have acquired secondary resistance to pazopanib (PazR) harbour an enrichment of phosphoproteins that play a role in the regulation of actin cytoskeleton dynamics (Fig. 3). These include the LIM domain family of proteins CRIP2, LASP1, MICALL1 and PDLIM7 which have previously been shown to be localised in focal adhesion complexes and play important roles in mechanotransduction signalling [46], [57], [58]. In addition, phosphoproteins that contain the CH domain, a 100 amino acid residue domain that binds to actin filaments, are similarly enriched in PazR cells [48], [49]. Published phosphoproteomic studies have found that melanoma cells with acquired resistance to BRAF inhibitors display elevated levels of phosphoproteins that function in cytoskeletal regulatory pathways [59], [60]. It remains to be determined if the upregulation of cytoskeletal pathways observed in our current study and in the previous melanoma reports is a cause or consequence of the acquisition of drug resistance. However given that this class of proteins is poorly explored as oncology drug targets [61], these phosphoproteomic studies provides a rich source of new candidates for target validation and drug development to overcome drug resistance. In contrast to the PazR cells, acquired resistance to dasatinib in the DasR subline leads to the upregulation of components of the insulin receptor/IGF-1R signalling pathway compared to parental A204 cells (Fig. 5). Activation of IGF-1R signalling is a well-established bypass mechanism of resistance to many kinase inhibitors including EGFR, HER2, MEK and BRAF inhibitors [62], [63], [64], [65], [66]. Furthermore, intrinsic resistance to dasatinib in a panel of non-small-cell lung cancer cell lines has been causally linked to the upregulation of Insulin-like growth factor (IGF)-binding protein-2 (IGFBP2) which acts as a carrier protein for the IGF ligands [67]. Our data suggests that the Insulin receptor/IGF-1R pathway is an actionable target for salvage therapy and further investigation to dissect the contribution of components of this pathway to acquired dasatinib resistance is planned.

One limitation of our study is the relatively modest number of phosphorylation sites identified in our analysis. We quantified ~ 7000 phosphorylation sites in our experimental dataset (Fig. 1C) which is comparable with published reports on single-shot unfractionated samples [42], [44], [45]. In addition, increased precursor ion complexity associated with SILAC labelling results in a decrease in unique phosphopeptide identification [68]. Greater depth of coverage in the phosphoproteome can be achieved with additional pre-fractionation steps [42], [44], [45], and combining orthogonal phosphopeptide enrichment strategies [69], [70]. Another limitation of the study is the focus on phosphoproteomic analysis without accounting for protein abundance changes. In the absence of a deep proteome analysis of the resistant and sensitive cell lines, we are unable to distinguish if the phosphorylation changes observed in our dataset are due to alterations in protein phosphorylation stoichiometry or at the level of total protein expression. Notwithstanding these limitations, our study demonstrates that candidate resistance signalling pathways can be readily identified with this approach.

Our phosphoproteomic analysis finds that acquired resistance to pazopanib and dasatinib leads to a 6.0% and 9.7% change, respectively, in the quantified phosphoproteome compared to parental A204 cells (Figs. 2A and 4A). A recent study by Nagata et al., showed that acquired resistance to the TKI imatinib in a GIST cell line displayed alterations in ~ 75% of the phosphoproteome when compared to the parental sensitive cell line [21]. In contrast, a phosphoproteomic analysis by Lee et al., of acquired resistance to the TKI lapatinib in a gastric cancer cell line showed that 5% of the phosphoproteome was significantly altered versus the parental cells from which resistance was derived [20]. The low percentage of phosphorylation changes observed in our study may be due to a number of factors. One reason could be that the depth of phosphoproteome coverage is less comprehensive in our analysis and that we are only sampling the most abundant phosphoproteins in the cell, although this is unlikely given that the study by Nagata et al., identified ~ 1000 phosphoserine/threonine sites with a 75% difference observed while Lee et al., quantified 6500 phosphosites with only 5% alterations seen. Another contributing factor is that the underlying genomic drivers of the cell lines used in the different studies are distinct. Unlike the GIST and gastric cell lines used in the previous studies, the A204 rhabdoid tumour cell line has a very simple genome where the loss of the SWI/SNF chromatin remodelling subunit SMARCB1 is the only known cancer-associated driver [12], [72], [73], [74]. It is plausible that loss of SMARCB1 may be sufficient to drive acquired TKI resistance with limited alterations in the phosphoproteome. Finally it is also possible that different TKIs reprogram cellular signalling networks to achieve drug resistance using distinct mechanisms [4], [75], [76].

The targeted drug profiling analysis identified the HSP90 inhibitor NVP-AUY-922 as a novel means to overcome pazopanib and dasatinib resistance (Fig. 7). The small molecule inhibitor panel that we employed was designed to block a range of distinct bypass pathways that have previously been associated with TKI resistance [24]. We show that AUY-922 is capable of not only overcoming acquired resistance in the form of salvage therapy, but also has utility when applied in the first-line setting (Fig. 7C). HSP90 inhibitors have been deployed as salvage therapy in clinical trials for TKI-resistant lung cancer and GIST with varying results [77], [78]. The rationale for this approach is based on pre-clinical evidence that cancer cells are dependent on HSP90 for stabilising client proteins such as TKI resistance-associated mutants and kinases responsible for driving bypass signalling in cancer cells [79], [80]. Consequently inhibition of HSP90 has the potential to simultaneously block multiple resistance mechanisms in the context of salvage therapy [80]. The mechanism for the activity of AUY922 in sensitizing the PazR and DasR cells and the specific client proteins involved in mediating drug sensitivity remain unclear and will be the focus of future studies.

In summary, we have performed a phosphoproteomic analysis to determine the signalling pathways associated with acquired resistance to pazopanib and dasatinib. We also demonstrate that PazR and DasR cells are sensitive to the HSP90 inhibitor NVP-AUY-922. This study provides a useful resource for future studies investigating the determinants of pazopanib and dasatinib resistance; and identifies a new therapeutic strategy of inhibiting HSP90 function for further evaluation as a means of overcoming pazopanib and dasatinib resistance and tumour recurrence in multiple cancer types.

The following are the supplementary data related to this article.

Fig. S1 Distribution of phosphosites in phosphoproteomic datasets. Pie charts to show (A) distribution of phosphorylated serines ‘Ser’, threonines ‘Thr’ and tyrosines ‘Tyr’ and (B) known or novel phosphorylated sites in the PazR/A204 and DasR/A204 experiments using PhosphositePlus database [30]. Fig. S1

Fig. S2 Overlap of identified phosphotyrosine sites in the Wong et al. 2016 dataset and the current IMAC/TiO_2_ study. Fig. S2

Table S1 Phosphoproteomic dataset of PazR vs parental A204. Table S1

Table S2 Phosphoproteomic dataset of DazR vs parental A204. Table S2

Supplemental Table 3 Chemical compounds used in small molecule drug screen. Supplemental Table 3

## Transparency document

Transparency document. Image 1
