# Supplementary figures and images for: Quantitative phosphoproteomic analysis of acquired cancer drug resistance to pazopanib and dasatinib
Source: J Proteomics. 2018 Jan 6;170:130–40. doi: 10.1016/j.jprot.2017.08.015 (PMC5673060; doi:10.1016/j.jprot.2017.08.015)

**A**

PazR/A204

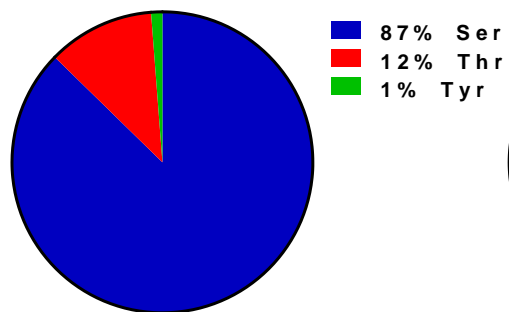

DasR/A204

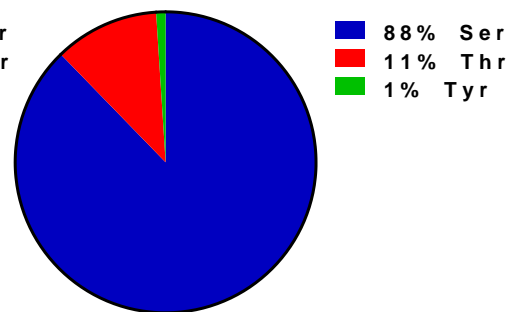

**B**

PazR/A204

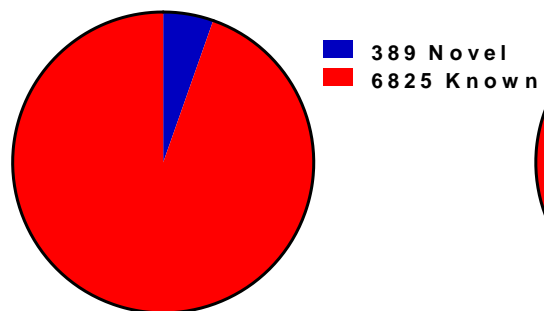

DasR/A204

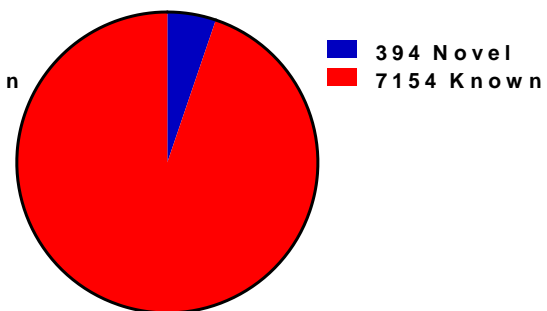

Supplement: Fig. S1 — Distribution of phosphosites in phosphoproteomic datasets. Pie charts to show (A) distribution of phosphorylated serines ‘Ser’, threonines ‘Thr’ and tyrosines ‘Tyr’ and (B) known or novel phosphorylated sites in the PazR/A204 and DasR/A204 experiments using PhosphositePlus database [30]. [file mmc1.pdf]

**PazR Wong et al**

**A204 PazR**

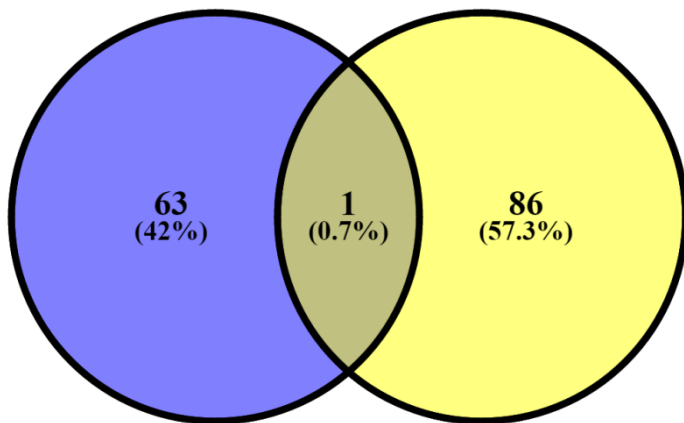

**DasR Wong et al**

**A204 DasR**

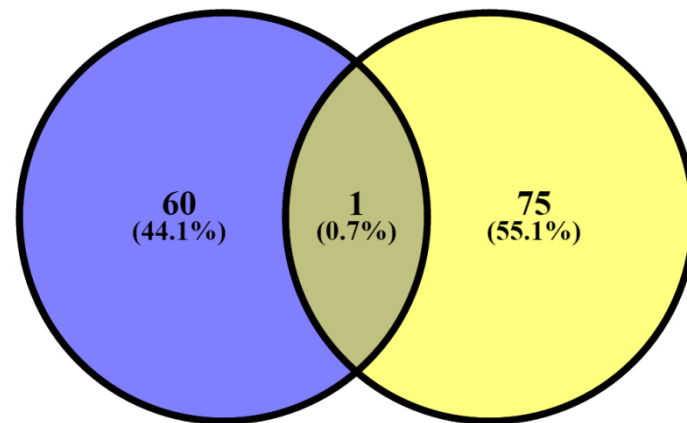

Supplement: Fig. S2 — Overlap of identified phosphotyrosine sites in the Wong et al. 2016 dataset and the current IMAC/TiO2 study. [file mmc2.pdf]
