# Supplementary material for: Quantitative phosphoproteomic analysis of acquired cancer drug resistance to pazopanib and dasatinib
Source: J Proteomics. 2018 Jan 6;170:130–40. doi: 10.1016/j.jprot.2017.08.015 (PMC5673060; doi:10.1016/j.jprot.2017.08.015)
Supplement: Supplemental Table 3 — Chemical compounds used in small molecule drug screen. [file mmc5.docx]

| **Supplemental Table 3 - Chemical compounds used in small molecule drug screen** | | |
| --- | --- | --- |
| Drug | Major Target(s) | Reference |
| Bosutinib | Src | [1] |
| Ponatinib | BCR-ABL, PDGFRα, FGFR | [2] |
| NVP-AUY922 | Hsp90 (BCR, ABL) | [3] |
| Neratinib | HER2 and EGFR | [4] |
| MK2206 | AKT | [5] |
| BEZ235 | PI3K/mTOR | [6] |
| JQ1 | BRD1-4 | [7] |
| AZD4547 | FGFR | [8] |
| BGJ398 | FGFR | [9] |
| AZD5363 | AKT | [10] |
| Dasatinib | BCR-ABL, SRC | [11] |
| Sunitinib | PDGFR, VEGFR, KIT | [12] |
| CCT244747 | CHK1 | [13] |
| Imatinib | BCR-ABL, KIT, RET, PDGFR | [14] |
| Pazopanib | VEGFR, PDGFRα, KIT | [15] |
| BX-795 | PDK1, TBK1, IKKe | [16] |
| Sorafenib | VEGFR, PDGFRα, RAF1, BRAF | [17] |
| Trametinib | MEK1, MEK2 | [18] |
| Palbociclib | CDK4, CDK6 | [19] |
| MRT67307 | TBK1, IKKe | [20] |
| Crizotinib | ALK, c-MET | [21] |
| DDR 1-in-1 | DDR1, DDR2 | [22] |
| PF562271 | FAK, PYK2, CDK1/2/3 | [23] |
| Lapatinib | HER2, EGFR | [24] |
| Rociletinib | EGFR T790M | [25] |
| AZD9291 | EGFR T790M | [26] |
| Ceritinib | ALK | [27] |
| Gefitinib | EGFR | [28] |

**Supplemental References**

[1] Boschelli DH, Ye F, Wang YD, Dutia M, Johnson SL, Wu B, et al. Optimization of 4-phenylamino-3-quinolinecarbonitriles as potent inhibitors of Src kinase activity. J Med Chem. 2001;44:3965-77.

[2] Noronha G, Cao J, Chow CP, Dneprovskaia E, Fine RM, Hood J, et al. Inhibitors of ABL and the ABL-T315I mutation. Curr Top Med Chem. 2008;8:905-21.

[3] Eccles SA, Massey A, Raynaud FI, Sharp SY, Box G, Valenti M, et al. NVP-AUY922: a novel heat shock protein 90 inhibitor active against xenograft tumor growth, angiogenesis, and metastasis. Cancer Res. 2008;68:2850-60.

[4] Rabindran SK, Discafani CM, Rosfjord EC, Baxter M, Floyd MB, Golas J, et al. Antitumor activity of HKI-272, an orally active, irreversible inhibitor of the HER-2 tyrosine kinase. Cancer Res. 2004;64:3958-65.

[5] Hirai H, Sootome H, Nakatsuru Y, Miyama K, Taguchi S, Tsujioka K, et al. MK-2206, an allosteric Akt inhibitor, enhances antitumor efficacy by standard chemotherapeutic agents or molecular targeted drugs in vitro and in vivo. Mol Cancer Ther. 2010;9:1956-67.

[6] Maira SM, Stauffer F, Brueggen J, Furet P, Schnell C, Fritsch C, et al. Identification and characterization of NVP-BEZ235, a new orally available dual phosphatidylinositol 3-kinase/mammalian target of rapamycin inhibitor with potent in vivo antitumor activity. Mol Cancer Ther. 2008;7:1851-63.

[7] Filippakopoulos P, Qi J, Picaud S, Shen Y, Smith WB, Fedorov O, et al. Selective inhibition of BET bromodomains. Nature. 2010;468:1067-73.

[8] Gavine PR, Mooney L, Kilgour E, Thomas AP, Al-Kadhimi K, Beck S, et al. AZD4547: an orally bioavailable, potent, and selective inhibitor of the fibroblast growth factor receptor tyrosine kinase family. Cancer Res. 2012;72:2045-56.

[9] Guagnano V, Furet P, Spanka C, Bordas V, Le Douget M, Stamm C, et al. Discovery of 3-(2,6-dichloro-3,5-dimethoxy-phenyl)-1-{6-[4-(4-ethyl-piperazin-1-yl)-phenylamin o]-pyrimidin-4-yl}-1-methyl-urea (NVP-BGJ398), a potent and selective inhibitor of the fibroblast growth factor receptor family of receptor tyrosine kinase. J Med Chem. 2011;54:7066-83.

[10] Davies BR, Greenwood H, Dudley P, Crafter C, Yu DH, Zhang J, et al. Preclinical pharmacology of AZD5363, an inhibitor of AKT: pharmacodynamics, antitumor activity, and correlation of monotherapy activity with genetic background. Mol Cancer Ther. 2012;11:873-87.

[11] Lombardo LJ, Lee FY, Chen P, Norris D, Barrish JC, Behnia K, et al. Discovery of N-(2-chloro-6-methyl- phenyl)-2-(6-(4-(2-hydroxyethyl)- piperazin-1-yl)-2-methylpyrimidin-4- ylamino)thiazole-5-carboxamide (BMS-354825), a dual Src/Abl kinase inhibitor with potent antitumor activity in preclinical assays. J Med Chem. 2004;47:6658-61.

[12] O'Farrell AM, Abrams TJ, Yuen HA, Ngai TJ, Louie SG, Yee KW, et al. SU11248 is a novel FLT3 tyrosine kinase inhibitor with potent activity in vitro and in vivo. Blood. 2003;101:3597-605.

[13] Walton MI, Eve PD, Hayes A, Valenti MR, De Haven Brandon AK, Box G, et al. CCT244747 is a novel potent and selective CHK1 inhibitor with oral efficacy alone and in combination with genotoxic anticancer drugs. Clin Cancer Res. 2012;18:5650-61.

[14] Buchdunger E, Zimmermann J, Mett H, Meyer T, Muller M, Druker BJ, et al. Inhibition of the Abl protein-tyrosine kinase in vitro and in vivo by a 2-phenylaminopyrimidine derivative. Cancer Res. 1996;56:100-4.

[15] Harris PA, Cheung M, Hunter RN, 3rd, Brown ML, Veal JM, Nolte RT, et al. Discovery and evaluation of 2-anilino-5-aryloxazoles as a novel class of VEGFR2 kinase inhibitors. J Med Chem. 2005;48:1610-9.

[16] Feldman RI, Wu JM, Polokoff MA, Kochanny MJ, Dinter H, Zhu D, et al. Novel small molecule inhibitors of 3-phosphoinositide-dependent kinase-1. J Biol Chem. 2005;280:19867-74.

[17] Hotte SJ, Hirte HW. BAY 43-9006: early clinical data in patients with advanced solid malignancies. Curr Pharm Des. 2002;8:2249-53.

[18] Gilmartin AG, Bleam MR, Groy A, Moss KG, Minthorn EA, Kulkarni SG, et al. GSK1120212 (JTP-74057) is an inhibitor of MEK activity and activation with favorable pharmacokinetic properties for sustained in vivo pathway inhibition. Clin Cancer Res. 2011;17:989-1000.

[19] Fry DW, Harvey PJ, Keller PR, Elliott WL, Meade M, Trachet E, et al. Specific inhibition of cyclin-dependent kinase 4/6 by PD 0332991 and associated antitumor activity in human tumor xenografts. Mol Cancer Ther. 2004;3:1427-38.

[20] Clark K, Peggie M, Plater L, Sorcek RJ, Young ER, Madwed JB, et al. Novel cross-talk within the IKK family controls innate immunity. Biochem J. 2011;434:93-104.

[21] Zou HY, Li Q, Lee JH, Arango ME, McDonnell SR, Yamazaki S, et al. An orally available small-molecule inhibitor of c-Met, PF-2341066, exhibits cytoreductive antitumor efficacy through antiproliferative and antiangiogenic mechanisms. Cancer Res. 2007;67:4408-17.

[22] Kim HG, Tan L, Weisberg EL, Liu F, Canning P, Choi HG, et al. Discovery of a potent and selective DDR1 receptor tyrosine kinase inhibitor. ACS Chem Biol. 2013;8:2145-50.

[23] Roberts WG, Ung E, Whalen P, Cooper B, Hulford C, Autry C, et al. Antitumor activity and pharmacology of a selective focal adhesion kinase inhibitor, PF-562,271. Cancer Res. 2008;68:1935-44.

[24] Xia W, Mullin RJ, Keith BR, Liu LH, Ma H, Rusnak DW, et al. Anti-tumor activity of GW572016: a dual tyrosine kinase inhibitor blocks EGF activation of EGFR/erbB2 and downstream Erk1/2 and AKT pathways. Oncogene. 2002;21:6255-63.

[25] Walter AO, Sjin RT, Haringsma HJ, Ohashi K, Sun J, Lee K, et al. Discovery of a mutant-selective covalent inhibitor of EGFR that overcomes T790M-mediated resistance in NSCLC. Cancer Discov. 2013;3:1404-15.

[26] Cross DA, Ashton SE, Ghiorghiu S, Eberlein C, Nebhan CA, Spitzler PJ, et al. AZD9291, an irreversible EGFR TKI, overcomes T790M-mediated resistance to EGFR inhibitors in lung cancer. Cancer Discov. 2014;4:1046-61.

[27] Galkin AV, Melnick JS, Kim S, Hood TL, Li N, Li L, et al. Identification of NVP-TAE684, a potent, selective, and efficacious inhibitor of NPM-ALK. Proc Natl Acad Sci U S A. 2007;104:270-5.

[28] Baselga J, Averbuch SD. ZD1839 ('Iressa') as an anticancer agent. Drugs. 2000;60 Suppl 1:33-40; discussion 1-2.
